# In Vitro and In Silico Toxicological Properties of Natural Antioxidant Therapeutic Agent *Azima tetracantha*. LAM

**DOI:** 10.3390/antiox10081307

**Published:** 2021-08-18

**Authors:** Palanisamy Prakash, Nisha Kumari, Ekambaram Gayathiri, Kuppusamy Selvam, Manikavali Gurunadhan Ragunathan, Murugesan Chandrasekaran, Munirah Abdullah Al-Dosary, Ashraf Atef Hatamleh, Ashok Kumar Nadda, Manu Kumar

**Affiliations:** 1 Department of Botany, Periyar University, Periyar Palkalai Nagar, Salem 636011, India; drprakas2014@gmail.com (P.P.); kselvambot@periyaruniversity.ac.in (K.S.); 2 Department of Radiology, Seoul National University Hospital, Seoul National University College of Medicine, Seoul 03080, Korea; nishak.chambyal88@gmail.com; 3 Department of Plant Biology and Plant Biotechnology, Guru Nanak College, Chennai 600042, India; gay3purush@gmail.com; 4 Department of Advanced Zoology and Biotechnology, Guru Nanak College, Chennai 600042, India; principal@gurunanakcollege.edu.in; 5 Department of Food Science and Biotechnology, Sejong University, Gwangjin-gu, Seoul 05006, Korea; chandrubdubio@gmail.com; 6 Department of Botany and Microbiology, College of Science, King Saud University, Riyadh 11451, Saudi Arabia; almonerah@ksu.edu.sa (M.A.A.-D.); ahatamleh@ksu.edu.sa (A.A.H.); 7 Department of Biotechnology and Bioinformatics, Jaypee University of Information Technology, Waknaghat, Solan 173234, India; ashok.nadda09@gmail.com; 8 Department of Life Science, College of Life Science and Biotechnology, Dongguk University, Seoul 10326, Korea

**Keywords:** antiseptic, antioxidant, anti-proliferative, anti-inflammatory, docking, hela, polyphenol, phytomolecules

## Abstract

Plant-derived antioxidants are a large group of natural products with the capacity to reduce radical-scavenging. Due to their potent therapeutic and preventive actions, these compounds receive a lot of attention from scientists, particularly pharmacologists. The pharmacological activities of the *Azima tetracantha* Lam. (*AT*) plant, belonging to the Salvadoraceae family, reported here justifies its traditional use in treating several diseases or disorders. This study aims to look at the propensity of certain plant compounds found in natural *AT* plant extracts that might play a critical role as a secondary metabolite in cervical cancer treatment. There is a shortage of information on the plant’s phytochemical and biological characteristics. Methanol (MeOH) solvent extracts of the dried *AT* plant were screened phytochemically. Its aqueous extract was tested for antioxidant, antiseptic, anti-inflammatory, and anticancerous properties. Absorption Distribution Metabolism and Excretion (ADME/T), Docking, and HPLC were also performed. In clinical treatment, the plant shown no adverse effects. The antioxidant activity was evaluated and showed the highest concentration at 150 µg/mL (63.50%). MeOH leaf extract of *AT* exhibited the highest and best inhibitory activity against *Staphylococcus aureus* (15.3 mm/1000) and displayed a high antiseptic potential. At a 200 µg/mL concentration, MeOH leaves-extract inhibited red blood cells (RBC) hemolysis by 66.56 ± 0.40, compared with 62.33 ± 0.40 from the standard. Albumin’s ability to suppress protein denaturation ranged from 16.75 ± 0.65 to 62.35 ± 0.20 inhibitions in this test, providing even more support for its favorable anti-inflammatory properties. The ADME/T studies were considered for a potential cancer drug molecule, and one of our compounds from MeOH extract fills the ADME and toxicity parameters. The forms of compound 4 showed a strong hydrogen-bonding interaction with the vital amino acids (ASN923, THR410, LEU840TRY927, PHE921, and GLY922). A total of 90% of cell inhibition was observed when HeLa cell lines were treated with 300 µg/mL of compound 4 (7-acetyl-3a^1^-methyl- 4,14-dioxo-1,2,3a,3a^1^,4,5,5a,6,8a,9b,10,11,11a-tetradecahydro-2,5a epoxy5,6a (methanooxymethano)phenaleno[1′,9′:5,6,7]indeno[1,7a-*b*]oxiren-2-yl acetate). The polyphenol compounds demonstrated significant advances in anticancer drug properties, and it could lead to activation of cancer cell apoptosis.

## 1. Introduction

Naturally, there is a dynamic balance between the number of free radicals produced in the body and the antioxidants to scavenge or quench them for the body’s protection against harmful effects [1]. Antioxidant components in plants are derived from constituent nutrients with a proven radical-scavenging property. Thus, plant-based medications may include flavonoids, polyphenols, and flavoproteins, in addition to alpha-tocopherol, ascorbate, carotenoids, and zinc. Additionally, some plants or combinations of herbs may function as antioxidants by scavenging superoxide or boosting superoxide dismutase activity at different tissue regions [2]. Bacteriological infections are widespread in immuno-cooperated patients with the substantial expense of care and can be lethal. The increasing percentage of infectious rehabilitation of clinical agents and their effect on managing contagious diseases have started to present an extraordinary health challenge [3]. The possibility of pandemic disease increases the risk of medicinal resistivity for highly drug-resistant invasive bacterial pathogens. The reality is that many antiseptic agents are often produced through differing pathways and may produce transmutations that cause the resistance. *Staphylococcus aureus* is the most commonly occurring methicillin-resistant impurity formed by clinical microorganisms. The development of bacterial confrontations with antimicrobial agents explains the quest to seek new antimicrobials therapy [4].

In recent decades, cancer was believed to be associated with inflammation. This impression has been waning for a lengthy period. However, recent years have witnessed a resurgence of interest in the inflammation–cancer link, fueled by many lines of research and culminating in a widely recognized paradigm [5,6]. In recent times, several studies have highlighted the importance of swelling in tumor conditions. The intact macrophage cells are responsible for recognizing and eliminating infectious pathogen and apoptotic cells by synthesizing various bio-active pro-inflammatory mediators that monitor constant microbe invasion and unusual reactive oxygen species (ROS) development. Recurrent pro-inflammatory moderators at the injured tissue area resulted in the irregular or dysfunctional activation of inflammations. It is worth noting that irregular pro-inflammatory mediator secretions are primarily for tissue injuries, the transformation of the cell, and cancer [7]. The positive control of deregulated inflammatory pathways is aided by the repression of increased ROS production, pro-inflammatory mediators, and anti-inflammatory mediator secretion [8]. Compounds with a solid propensity to function as antioxidants and anti-inflammatory factors provide further benefits; in other words, an overabundance of inflammatory response will favor irregular ROS formation and cellular transition. Thus, phytoconstituents with multi-therapeutic properties in chronic diseases, including inflammation and cancer, play a vital role [9]. These phytoconstituents have multi-step action without side effects because the plant produces them as a normal response (secondary metabolite). Indeed, the previous report suggests the naturalistic phytoconstituents are successful in fighting against cancer [10].

Cancer is an uncontrolled growth of uncharacteristic cells, which can spread and appear in different body tissues, and sometimes if unnoticed, may cause mortality. It is a major problem disturbing people’s health worldwide [11]. Tumor cells perpetuate the disparate normal cells that use specific switches for controlling cellular growth and proliferation. Thus, identifying cancer cells targets, active sites, and revealing the target-lead interactions is vital for scheming new and effective therapeutics for tumors.

In the 21st century, the flare-up of diseases and discovery of protecting agents that deal via recognized, proposal, amalgamation, and resulting clinical trials poses actual challenges. In the past, drug design entailed a single or a few biological entities to curtail the study time and achieve high specificity and selectivity to avoid side effects arising from mistargeting [12,13]. Fortunately, computer-aided design and drafting (CADD) systems and recent state-of-the-art systematical tools have proven to be very beneficial for quickly judging the active molecules and extensive organic records [14,15]. These systems accelerate the precise identification of hopeful applicants before the start of any extensive chemical synthesis, biological testing (in vivo or in vitro), and clinical trials. Furthermore, it reduces cost and time [16,17]. The role of systematic tools in pharmacodynamics and pharmacokinetics is vital for the success of drug development. It cannot be ignored because properties such as solubility, hydrophobic effect, bioavailability, absorption, metabolism, and toxicity of the lead compounds are the parameters used to decide how good and safe the drugs are [18,19]. *AT* has been traditionally used for many diseases, including renal disease. It belongs to the Salvadoraceae family and is known as mulchangu in Siddha and kundali in Ayurveda [20]. The present investigation aims to design and screen molecules of five derivatives as a potential multi-target drug against cancer pharmacokinetics. In this study, we have used a natural antioxidant therapeutic agent, *Azima tetracantha* extract, that displays potential antiseptic and anticancerous properties. In addition, we believe that the target drugs screened herein could be potential therapeutics for bacterial infection and cervical cancer treatment.

## 2. Materials and Methods

### 2.1. Plant Material Collection and Extraction

The *Azima tetracantha* Lam. was obtained from Athamangalam, Nagapattinam Distt, Tamilnadu, India, from August to September 2018. The herbarium was maintained in the Department of Botany, Annamalai University (Plant authentication number AUBOT 262). One hundred grams of crushed leaf materials were extracted in a soxhlet apparatus for 8 h with solvent MeOH (99.35%, Sigma Aldrich, Bangalore, India). Later the extracts were sieved, and the solvent was evaporated by a rotatory evaporator (Heidolph, Germany) under a concentrated temperature at 40 °C, and the extracts were kept at 4 °C for further examination.

### 2.2. DPPH Radical-Scavenging Assay

Antioxidants scavenge the 2,2-Diphenyl-1-picrylhydrazyl (DPPH) radical by donating a proton, forming the reduced DPPH, and were evaluated by using the methodology [21]. Various concentrations (50, 75, 100, and 150 µg/mL) of the sample (4.0 mL) were mixed with 1.0 mL of a solution containing the DPPH radical (Sigma Aldrich, Bangalore, India), resulting in the final concentration of DPPH being 0.2 mM. The mixture was shaken vigorously and left to stand for 30 min, and the absorbance was measured at 517 nm. Ascorbic acid (20% Sigma Aldrich, Bangalore, India) was used as a positive control.

### 2.3. Evaluation of Antiseptic Properties

The antiseptic properties of methanol extract were tested against seven clinical bacterial strains (Gram + Ve: *S. aureus, B. cereus, B. subtillis,* and Gram-Ve: *S. typhi, K. pneumoniae, E. coli*). Bacteria were grown in a pure culture on a Luria broth medium. Using a cotton swab, the bacterial strains were placed onto agar plates. Next, the leaf extract was sprayed onto sterile discs with deionized chloramphenicol (Sigma Aldrich 98%, Bangalore, India), which serves as a positive control. The plates were then incubated overnight (35 °C) before being weighed on a standard scale [22].

### 2.4. Inhibition of Albumin Denaturation

In this procedure, an inhibitor of the albumin denaturation strategy was adapted with minor changes [23]. A 0.05 mL of distilled water and 0.45 mL of bovine serum albumin (5% aqueous solution, Sigma Aldrich, Bangalore, India) were used to make the reaction blend (0.5 mL; pH 6.3). A minimal volume of 1 N HCl (36% HiMedia, Mumbai, India) was used to adjust the pH to 6.3. Various plant extract concentrations were added to the reactants and incubated at 37 °C for 20 min before being heated at 7 °C for 5 min. After cooling the samples, 2.5 mL of phosphate buffer (Sigma Aldrich, Bangalore, India) saline was added. At 600 nm, turbidity was calculated spectrophotometrically.

(1) $$\mathrm{Percentage}\;\mathrm{Inhibition}\;(\%)=\frac{\mathrm{AbsControl}-\mathrm{AbsSample}}{\mathrm{AbsControl}}\times 100$$

### 2.5. HRBC Membrane Stabilization Method

Blood (2 mL) was obtained from healthy volunteers (From Rajamuthaia Medical college, Chidambaram, India on 20 July 2020) and diluted with an equivalent amount of sterile Alsevers solution (2% dextrose, 0.8% sodium citrate, 0.5 % citric acid, and 0.42% NaCl in purified water; HiMedia, Mumbai, India) and centrifuged at 3000 rpm. The loaded cells were cleaned with a saline solution and stored undisturbed at 4 °C in the 10% *v*/*v* saline. Various herbal extracts (62.5, 125, 250, 500 mg/mL) and Aspirin (as a regular and positive control agent, distilled water used rather than hypo saline to achieve 100% hemolysis) were added then combined with 2 mL of hyposaline, 1 mL of phosphate buffer, and 0.5 mL of 10% HRBC suspension (Sigma Aldrich, Bangalore, India). The blends were incubated at 37 °C for 30 min, and then centrifugation for 20 min at 3000 rpm, and the supernatant hemoglobin solution was analyzed spectrophotometrically at 560 nm [24].

(2) $$\;\mathrm{Stabilization}\;(\%)=\frac{\mathrm{AbsControl}-\mathrm{AbsSample}}{\mathrm{AbsControl}}\times 100$$

### 2.6. HPLC Analysis

#### 2.6.1. Extraction Solvent

A mixture of alcohol, water, and hydrochloric acid (50:20:8) was prepared. Subsequently, a methanol, water, and phosphoric acid (100:100:1) mixture was prepared for the mobile phase.

#### 2.6.2. Standard Solutions

Accurately weighed quantities of Rutin (94%, Sigma Aldrich, Bangalore, India) were transferred to separate volumetric flasks. Each was dissolved quantitatively with MeOH to obtain a standard solution of 1 mg per mL, respectively.

#### 2.6.3. Test Solution

About 10.0 g of the given sample was finely powdered, accurately weighed, and transferred to a 250 mL flask fitted with a reflux condenser. Next, 78 mL of extraction solvent were added and refluxed on a hot water bath for 135 min after cooling at room temperature and transferred to a 100 mL volumetric flask. Finally, 20 mL of MeOH was added to the 250 mL flask and sonicated for 30 min. After filtration, the filtrate was collected in a 100 mL volumetric flask. The residue on the filter was washed with MeOH, and it is collected in the same 100 mL volumetric flask.

#### 2.6.4. Chromatographic System and Procedure

The liquid chromatography is equipped with a 270 nm detector and a 4.6-mm × 25-cm L1 Octadecyl silane column. The flow rate is about 1.5 mL per minute. Standard solution 1 was chromatographed, and the peak responses were recorded. About 20 µL of the standard solutions with an equal volume of test solution were injected into the chromatograph separately. Chromatograms were recorded for the measurement of the areas for the major peaks.

### 2.7. GC-MS Analysis

Gas chromatography-mass spectrometry (GC-MS) was performed on an *Azima tetracantha* MeOH sample (“JEOL GCMATE II GC-MS-Agilent 6890 N Network GC” system). Two milliliters of active fractions were dissolved in HPLC grade MeOH (Sigma Aldrich, Bangalore, India) before being exposed to MS and GC on a JEOL GC mate fitted with the secondary electron multiplier. The column (HP5) was made of fused silica and had a 50 mm_0.25 mm I.D. The experiment lasted 20 min at 100 °C. The temperatures of the column and injector were fixed to 235 and 240 °C, respectively. Helium was used as the carrier gas in this protocol, with a split ratio of 5:4. In a splitless injector, a sample volume was evaporated with 2 mL. The run time was 22 min at 300 °C, and the detection of the compound was accomplished using GC combined with mass spectrometry. An established specification was run concurrently for reference. The test fraction’s molecular weight, mathematical formula, compound composition, and peak area were quantified by comparing them to established compounds in the spectral library (NIST 05).

### 2.8. Computational Analysis

#### 2.8.1. Drug-Likeness Studies

All the molecules studied were analyzed in silico for their molecular properties and drug-likeness limits. It was constructed using hypothetical approaches with the aim of identifying molecules that meet the ideal criteria for exhibiting as drug-like molecules as described by Lipinski’s rule of five [25], and other physicochemical parameters were considered using the Biovia DS vs. 4.5 software module “Calculation of Molecular Properties”. Additionally, specific properties were predicted using open web-based resources, such as Molsoft (http://www.molsoft.com/, accessed on 15 February 2021) and the computer app Molinspiration.

#### 2.8.2. Swiss ADME/Toxicity

ADME (Absorption, Distribution, Metabolism, Excretion) and Toxicity of the calculated compounds were predicted along with a massive database on the swiss ADME/T web server (http://www.swissadme.ch/, accessed on 16 February 2021), and the server can hypothesize high-precision therapeutic and medicinal properties [26].

#### 2.8.3. Molecular Docking

The construction of the compounds and proteins was prepared as per the prearrangement. Auto Dock vina 4.2 was selected for docking studies by the standard procedure [27]. The amalgams originated were established into a 3D model. A 2D display of the target compounds was performed to verify their structures and the formal charges of their atoms. Then, the energy minimization was extended to all conformers. The minimization processes were carried out until a 0.01 kcal mol 1 root mean square deviation (RMSD) gradient and the 0.1 AR MMFF94X force field distance were reached. Then, the partial expenditures were determined automatically. The database was saved as MDB- (Mongo, Database) for further docking calculations.

#### 2.8.4. Enrichment and Network Analysis

STRING (https://string-db.org, accessed on 12 August 2021) was used to search the list of possible targets [28]. A database was created for organic evolution, molecular role, and cellular mechanisms, and a protein interaction enrichment study was conducted. Further, the potentially moderated pathways were indented in relation to the KEGG pathway record. The network between plants, their phytoconstituents, changed proteins, and organized pathways were hypothesized. The network was viewed as oriented, and the node size was set to “low values to tiny sizes,” node color was set to “low values to bright colors,” and the whole structure was examined using edge count.

### 2.9. Cell Line

The HeLa cervical cancer cell lines were collected from the “National Centre for Cell Science” (NCCS) Pune, India. The HeLa cell lines were cultured in “Dulbecco’s Modified Eagle’s Medium” (DMEM) supplemented with 10% fetal bovine serum (Sigma Aldrich, Bangalore, India) (FBS) and antimycotic and antibiotic reagent (1%) at 37 °C in the presence of 5% moistened CO_2_.

### 2.10. Cell Viability Assay by Enzyme-Linked Immunosorbent

The cell viability was evaluated using the 3-(4, 5-dimethylthiazol-2-yl) -2,5-diphenyl tetrazolium bromide (MTT, Sigma Aldrich, Bangalore, India) assay. Briefly, HeLa cells in the log phase at a concentration of 1.0 × 10^4^/well were cultured in 96-well plates and incubated in 5% CO_2_ at 37 °C for 24 h. Throughout the cultivation, the cells were constantly treated by the addition of MeOH at varying concentrations (18.75, 37.5, 75, 150, and 300 g/mL). The control group was kept under the same conditions with 0.1% dimethyl-sulfoxide (DMSO, Sigma-Aldrich, Bangalore, India). After incubation for 24 h, 10 µL of 5 mg/mL MTT was added to each well and incubated for an additional 4 h. Then, the supernatant was discarded, and 100 µL of DMSO was added to each well, and the 96-well plates were vortexed for 10 min. An enzyme-linked immunosorbent assay (ELISA) reader was used to record the optical density (OD, Sky technology, Ahmedabad, Gujarat, India) at 570 nm.

### 2.11. Statistical Analysis

The experiments were carried out three times. The findings were analyzed by SD, mean assays, and they were subjected to One-Way ANOVA, Dunnett’s multiple evaluation experiments, and PRISM program version 5.2. (Graph Pad Software Inc, San Diego, California, USA).

## 3. Results

### 3.1. Azima Tetracantha Exhibits Antioxidant Properties

**Figure 1:**
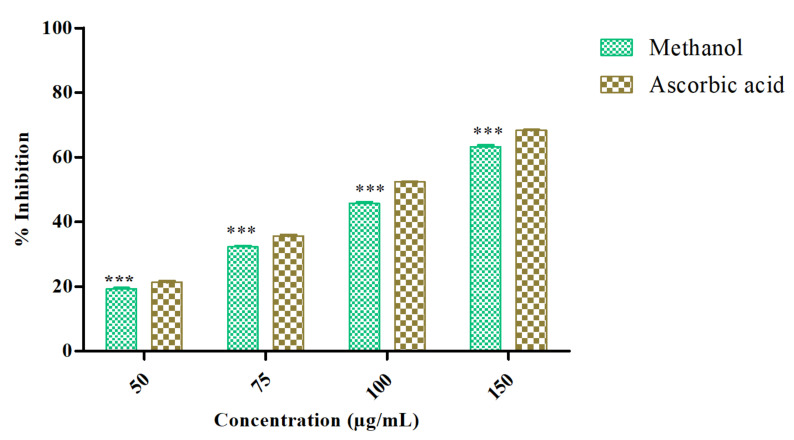
DPPH radical-scavenging ability of methanol extract of *Azima tetracntha. The values are* Mean ± SD. One-way ANOVA was used to analyze the significant difference among groups. The symbol (***) indicate the significant difference (*p <* 0.001).

The antioxidant activity of *Azima tetracantha* was determined by its DPPH free-radical-scavenging ability. The DPPH radical-scavenging activity of MeOH leaf extract was evaluated and compared with positive control ascorbic acid. The percentage of inhibition (% inhibition measured at various concentrations as (50, 75, 100, and 150 µg/mL) of the sample as well as standard were measured (Figure 1). The highest concentration was found at 150 µg/mL (63.50%), which was followed by 50 µg/mL (22.11%) as sample inhibition values, and the highest concentration of standard was 50 µg/mL (68.35%) and the lowest was 50 µg/mL (26.50%). Polyphenolic compounds are considered to be very important plant constituents that are responsible for free radical-scavenging ability owing to their hydroxyl groups.

### 3.2. Antiseptic Properties of Azima Tetracantha

The antiseptic properties of MeOH of the leaf of *Azima tetracantha* against bacterial pathogens and results are presented in (Table 1).

**Figure 2:**
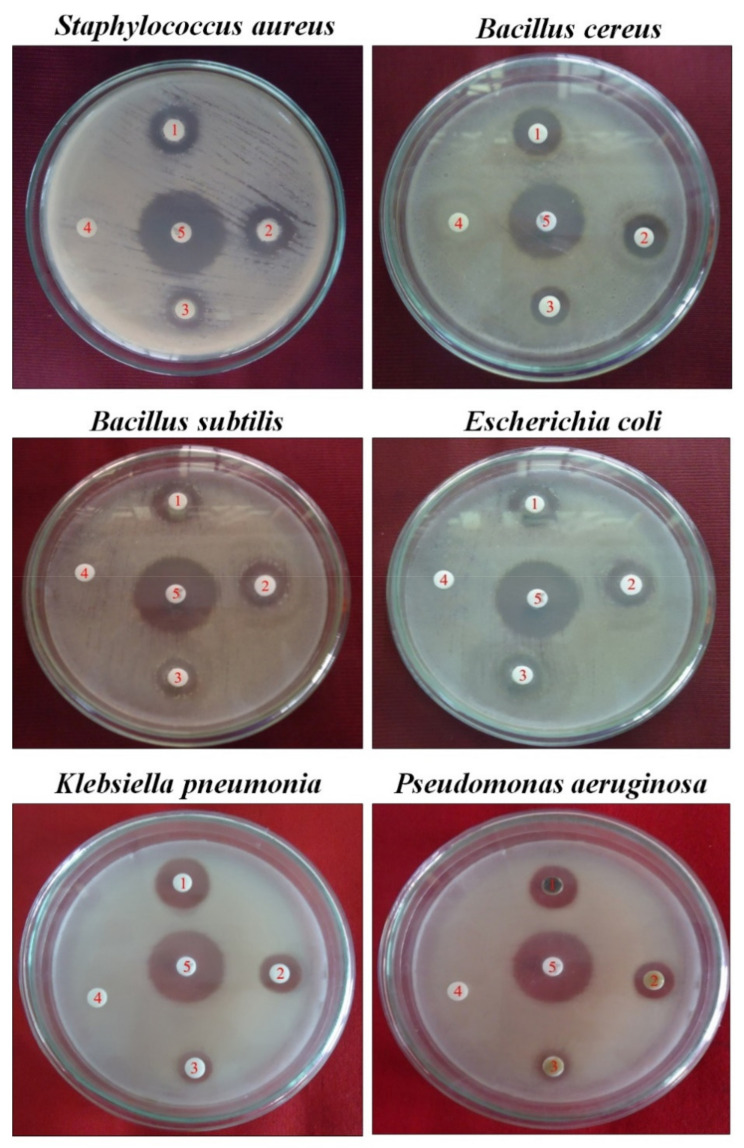
Antiseptic activity is indicated as a zone of inhibition by MeOH leaf extract of *Azima tetracantha* Lam. Numbers in the Petri dish are as follow: (1) 500, (2) 125, (3) 50 µg/disc, (4) negative control DMSO, and (5) positive control chloramphenicol.

The minimum zones of inhibition for MeOH ranged from 8.2 to 15.3 mm. The methanol extract of the *AT* leaf exhibited the highest and best inhibitory activity against *Staphylococcus aureus* (15.3 mm/500 µg/disc) (Figure 2). Thus, the screenings of *AT* medicinal plants indicate their antibacterial properties.

### 3.3. In Vitro Anti-Inflammatory Activity

#### 3.3.1. Inhibition of Albumin Denaturation

**Figure 3:**
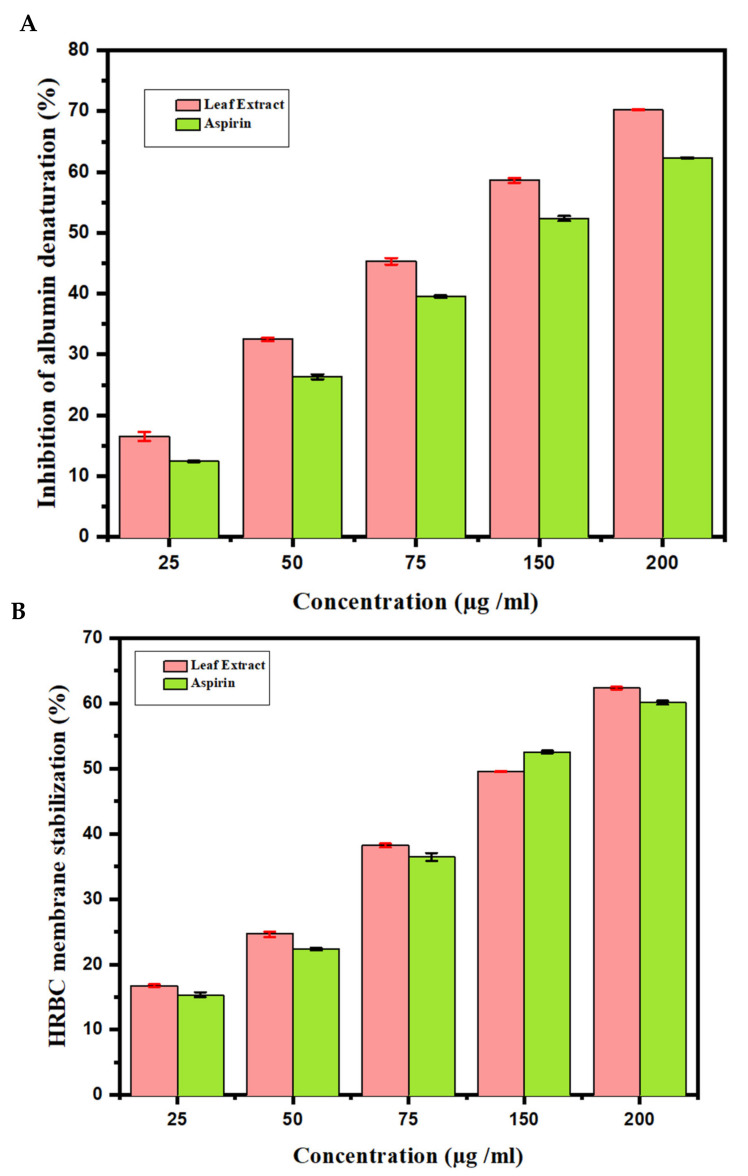
Anti-inflammatory activity of *Azima tetracantha*. (**A**) Protein denaturation by using egg albumin. (**B**) Human red blood cell (HRBC) membrane stabilization of assay. Aspirin is used as a standard drug.

The effects of anti-inflammatory behavior assessed towards the denaturation of egg albumin are outlined (Figure 3A). This figure describes the observations of in vitro flow cytometry of the anti-inflammatory properties assessed against the denaturation of egg albumin. At a concentration of 200 μg/mL, the highest inhibition obtained was 67.20 ± 0.10. Aspirin used as a standard drug showed a 60.69 ± 0.10 inhibition at a 200 μg/mL concentration. In addition, the results of the anti-inflammatory examination for the human red blood cell membrane were revealed. The *AT* MeOH extract produced a 62.56 ± 0.40 inhibition of RBC hemolysis at a 200 μg/mL concentration, compared with 62.33 ± 0.40 produced as standard by the standard drug Aspirin (Figure 3B). From the data, it can be concluded that MeOH leaf extract of *AT* showed a greater response than Aspirin.

#### 3.3.2. GC-MS Analysis of *Azima tetracantha*

The findings of GC-MS analysis of *AT* MeOH extract led to the identification of a number of chemicals. Five major peak compounds (3,4-Dimethyl-5-oxo-2,5-dihydro-1H- pyrrol-2-yl)-[4,4-dimethyl-5-[2,3,3-trimethyl-5-methylthio-3,4-dihydro-2H-pyrrolyl methylene] pyrrolidin-2-ylene] -thioacetic acid, S-[tert-butyl] ester (12.15%), 1H-Inden- 1-one,2,3-dihydro-5,6-dimethoxy-3-methyl (12.65%), 5-(p-Aminophenyl)-4-(O-tolyl)-2- thiazolamine (14.27%), 92-(3-acetoxy-4,4,10,13,14 pentamethyl 2,3,4,5,6,7,10,11,12,13,14,15,16,17-tetradecahydro-1H-cyclopenta[a]phenanthren-17-yl) propanoic acid (16.05%), and 3,9a;14,15-Diepoxypregen-16-en-20-one,3,11a;18-triacetoxy (23.24%) were present in the MeOH extracts of *AT*. The existence of multiple components with varying retention periods was verified by the GC-MS spectra (Figure S1). The mass spectrometer examines the chemicals eluted at various intervals to determine their type and structure. The big compound breaks into tiny compounds, causing peaks with various m/z ratios to emerge. These mass spectra represent the compound’s fingerprint, which may be recognized using the data library.

#### 3.3.3. High-Performance Liquid Chromatography Analysis

HPLC analysis of the MeOH leaf extract of *AT* showed that several peaks correspond to flavonoids at 650 nm. The flavonoid presence was validated by contrasting the samples’ retention period to the Rutin and comparing Rutin’s resulting peak heights in the methanol extract (Figure S2). The sample chromatograms revealed many peaks that did not correlate to the flavonoid criteria we used, mainly at retention times ranging from 2.071 to 8.814 min. These peaks may be flavonoid peaks. The ascorbic acid, gallic acid, and resorcinol found in the extract will act synergistically in metabolizing the biological activities. Polyphenol compounds have made significant advances in anticancer drug production, with the ability to kill cancer cells by inducing apoptosis.

#### 3.3.4. Scrutiny of Pharmacokinetic Properties

The lead molecules, which may be a potential inhibitor, should have desirable pharmacokinetics to pass initial clinical trials. Thus, the first screening of the lead molecules was performed based on their physicochemical or ADME/T properties. All of the five derivatives passed this test. The five derivatives (Table 2, Table 3 and Table 4) present that the lead molecules with physicochemical and pharmacokinetic properties only have high scores against the five targets and active molecules.

### 3.4. Molecular Docking

**Figure 4:**
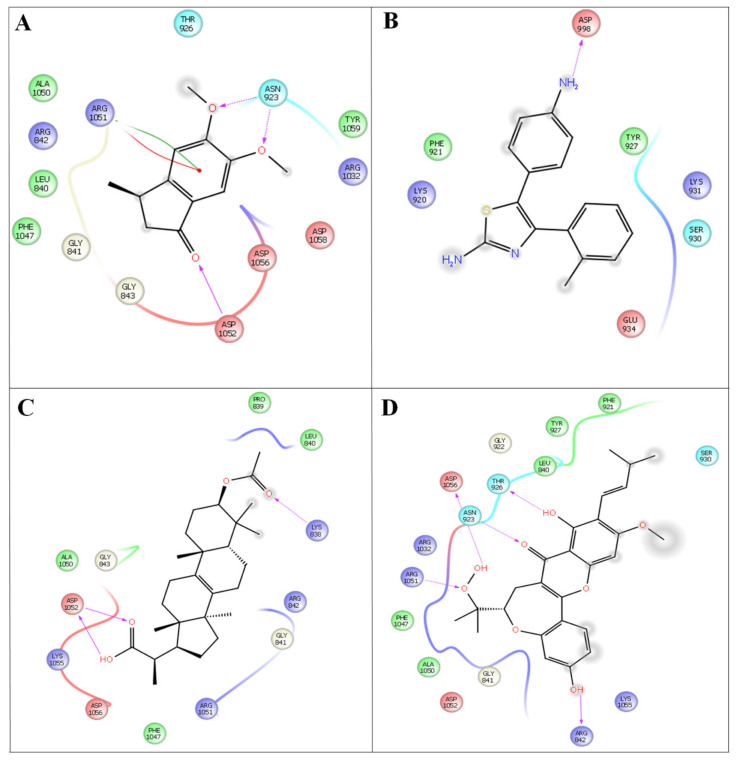
In silico docking studies representing 2 dimentional *Azima tetracantha* complexes with ligand interaction. (**A**) Compound 1, [5,6-dimethoxy-3-methyl-2,3-dihydro-1H-indene-1-oneintertaction 4AGD]. (**B**) Compound 2, [5-(4- aminophenyl)-4-(o-tolyl) thiazol-2-amine]. (**C**) Compound 3, [2-(3-acetoxy-4,4,10,13,14-pentamethyl- 2,3,4,5,6,7,10,11,12,13,14,15,16,17-tetradecahydro-1H-cyclopenta[a]phenanthren-17-yl) propanoic acid]. (**D**) Compound 4, [7-acetyl-3a1-methyl-4,14-dioxo1,2,3a,3a1,4,5,5a,6,8a,9b,10,11,11a-tetradecahydro-2,5a1-epoxy5,6a(methanooxymethano)phenaleno[1′,9′:5,6,7]indeno[1,7a-b]oxiren-2-yl acetate].

Molecular docking was performed for the two protein targets with all the inhibitors (leads) to identify ligand binding sites and binding modes, followed by the best scores (Figure 4).

The discovery of multi-target drugs is a hot topic, as studies revealed that multi-target drugs have a safer therapeutic profile and applications to more complex diseases. The detailed intermolecular interactions and binding energy values of the molecules with VEGFC and EFGR are listed in Table 5.

**Figure 5:**
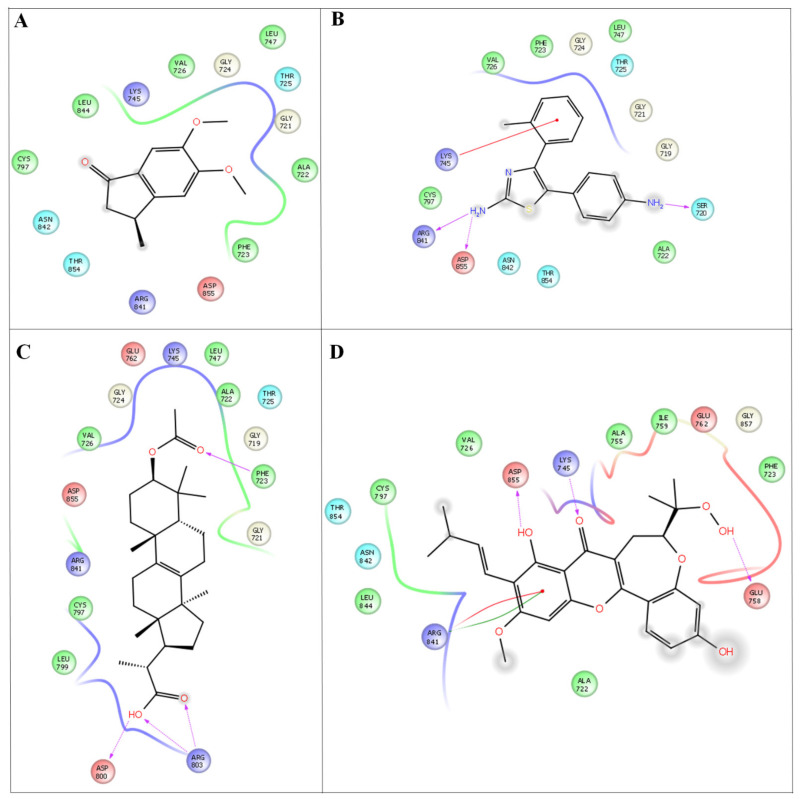
In silico molecular docking studies representing 2 dimentional *Azima tetracantha* complexes with ligand interaction. (**A**) 6-dimethoxy-3-methyl-2,3-dihydro-1h-indene-1-one interaction 2ITY Ligplot. (**B**) 5,6-dimethoxy-3- methyl-2,3-dihydro- 1H-indene-1-one. (**C**) 2-(3-acetoxy-4,4,10,13,14-pentamethyl-2,3,4,5,6,7,10,11,12,13,14,15,16,17- tetradecahydro-1H-cyclopenta [a]phenanthren-17-yl) propanoic acid. (**D**) 7-acetyl-3a1-methyl-4,14-dioxo- 1,2,3a,3a1,4,5,5a,6,8a,9b,10,11,11a-tetradecahydro-2,5a1-epoxy5,6a (methanooxymethano)phenaleno[1′,9′:5,6,7] indeno [1,7a-b]oxiren-2-yl acetate.

Among the four compounds, the compounds 37-acetyl-3a^1^-methyl-4,14 dioxo1,2,3a,3a1,4,5,5a,6,8a,9b,10,11,11atetradecahydro2,5a1epoxy5,6a (methanooxymethano)phenaleno[1′,9′:5,6,7]indeno[1,7a-b]oxiren-2-yl acetate and (2-(3- acetoxy-4,4,10,13,14-pentamethyl-2,3,4,5,6,7,10,11,12,13,14,15,16,17-tetradecahydro-1H cyclopenta [a]phenanthren-17-yl) propanoic acid, 5-(4-aminophenyl)-4-(o-tolyl) thiazol-2-amine and 5,6-dimethoxy-3-methyl-2,3-dihydro-1H-indene-1-one) exhibit both anti-cancer and anti-inflammatory properties. Compounds 4 (−52.25 kcal/mol), 3 (−39.05 kcal/mol), 2 (−26.65 kcal/mol), and 1 (26.59 kcal/mol) have better binding affinities and are well docked with the EFGR enzyme. Compound 4 (−44.60 kcal/mol) gives the highest binding energy values compared to the other compounds (compound 1 (34.3 kcal/mol), compound 2 (−36.51 kcal/mol), and compound 3 (−44.53 kcal/mol)) (Figure 5). As a result, the newly developed compounds were proven to have excellent natural accessibility, indicating that they may be readily produced in the laboratory.

Compound 4 forms a strong hydrogen-bonding interaction with the vital amino acids (ASN923, THR410, LEU840, TRY927, PHE921, and GLY922) compared with compound 3. The stabilization of compound 4 in VEGFC’s active site is dependent on these interactions. With a combination of growth factors and hypoxia, oncogene expression upregulates VEGF, a central mediator of angiogenesis in cancer. They are also hemorrhagic and leaky, resulting in a robust interstitial strain.

### 3.5. Protein–Protein Interaction (PPI) Network Analysis

**Figure 6:**
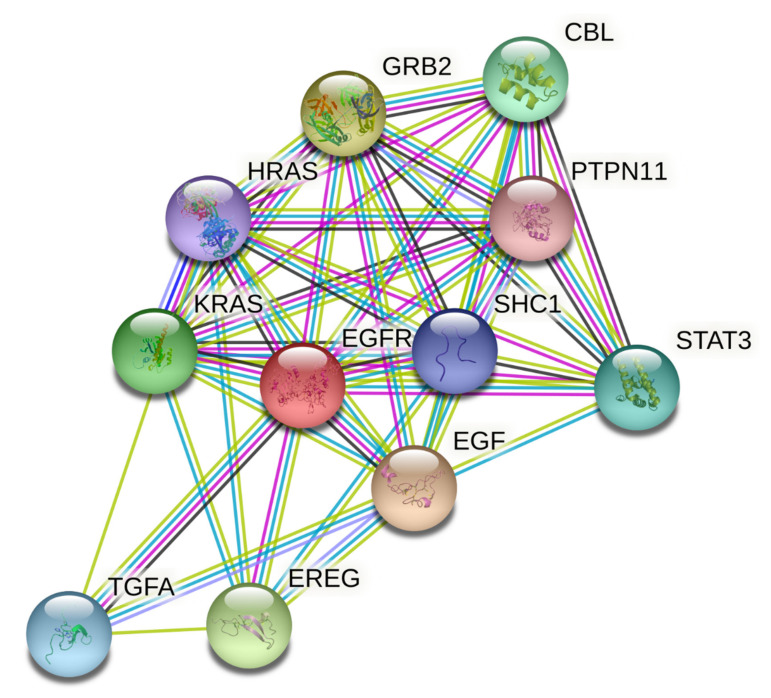
Protein–protein interaction network of targets related to cancer. The colored nodes represent candidate proteins, and colored lines represent protein interactions. Light green—represents text mining; Black—represents co-expression; Light blue—represents known interactions from curated databases; State blue—represents protein homology; Magenta—represents experimentally determined known interactions; Green—represents predicted interactions between neighborhood genes; Red—represents predicted interactions of gene fusion; and Blue—represents predicted interactions between co-occurred genes.

Thirty-one primary hub nodes were selected and inserted into the STRING database, establishing a connection between two distinct nodes (proteins/genes). The PPI network (21 edges and 11 nodes) was constructed (Figure 6) and may play a key role in oncology’s pharmacological impact phase.

Three targets, EGFR, EPGN, and BTC, did not communicate with each other. High trust is described as a rating greater than or equal to 0.998. EREG, EGF, TGFA, and GRB2, as well as HRAS, were two separate nodes with strong trust levels.

### 3.6. Molecular Analysis

At the statistical level, the *p*-value was <0.65. AT was mostly associated with the MF 34 and led to biological quality control, prostate gland growth, control of prostate epithelial cell proliferation found by transmission, and gene-set enrichment analysis knowledge along with a gene analysis set improvement for these 122 genes. EREG, EGF, TGFA, GRB2, and HRAS proteins have common biological functions as well molecular functions (specifically the characteristics of protein homodimerization). The analysis of drug aims is among the most recognized techniques for systematically discovering medicines based on ligands. Our bio-active products were found to be exposed to -acetyl-3a^1^-methyl-4,14-dioxo-1,2,3a,3a^1^,4,5,5a,6,8a,9b,10,11,11a-tetradecahydro-2,5a^1^nepoxy5,6a(methanooxymethano)phenaleno[1′,9′:5,6,7] indeno [1,7a-*b*]oxiren-2-yl acetate (ASN923, THR410, LEU840TRY927, PHE921, GLY922). The dock complex exchanges for each ligand were analyzed and inspected with efficient remains.

### 3.7. KEGG and REATOME Pathways Enrichment Analysis

A total of 21 KEGG pathways (*p*-value < 0.05) have been mapped into active compound objectives. The results showed that EREG, EGF, TGFA, GRB2, and HRAS were substantially enhanced throughout the pathway. The number of target mapping pathways was higher than ErbB signaling pathways in cancer (hsa04012 number = 9), non-small cell lung cancer (hsa05223, number = 7), and EGR Tyrosine kinase inhibitors (hsa05219, number = 4). The objectives of active compounds were mapped to twenty-one respite pathways (*p*-value < 0.05). The findings showed a substantial rise in EREG, EGF, TGFA, and GRB2 in paths. The mapping target paths were greater than the signaling paths (HSA 180336, number = 5, HSA5638303 number = 2, HSA179812, number = 4, HSA212718 number = 2, and HSA6802953 number = 2).

### 3.8. Compound-Target-Pathway Interaction Network

The *AT* compounds not only work on the same target protein but have been used on various target proteins through several pathways, reflective of the synergistic effect of AT “various components and numerous objectives”. Finally, our results reveal novel structures with similarly functional structural features and two compounds with higher binding energy than the active molecules. The active site residual was mapped on the binding site, and the extracts were identified in each target structure in the dynamic field. The analysis of molecular structures is the most effective method to evaluate the molecular mechanisms of the drug’s action. Our findings will also finally demonstrate that the novel structures with comparable structural features with similar activities are identified concerning the most significant binding energy compounds inactive molecules.

### 3.9. Compound Induce an Anti-Proliferative Effect on Cervical Cancer Cell Line

**Figure 7:**
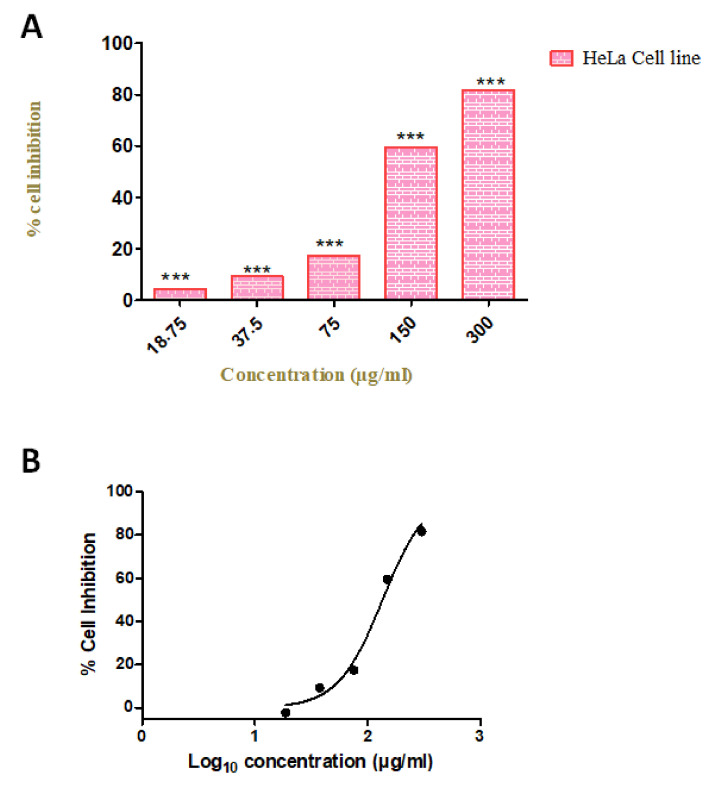
Cellular metabolic activity indicated by cell viability. (**A**) Cytotoxicity assays of *Azima tetracantha* compound treated for 24 h in HeLa cell lines. The symbol (***) indicate the significant difference (*p <* 0.001). (**B**) Percentage cell inhibition of Log_10_ concentration (µg/mL) on HeLa cell lines.

**Figure 8:**
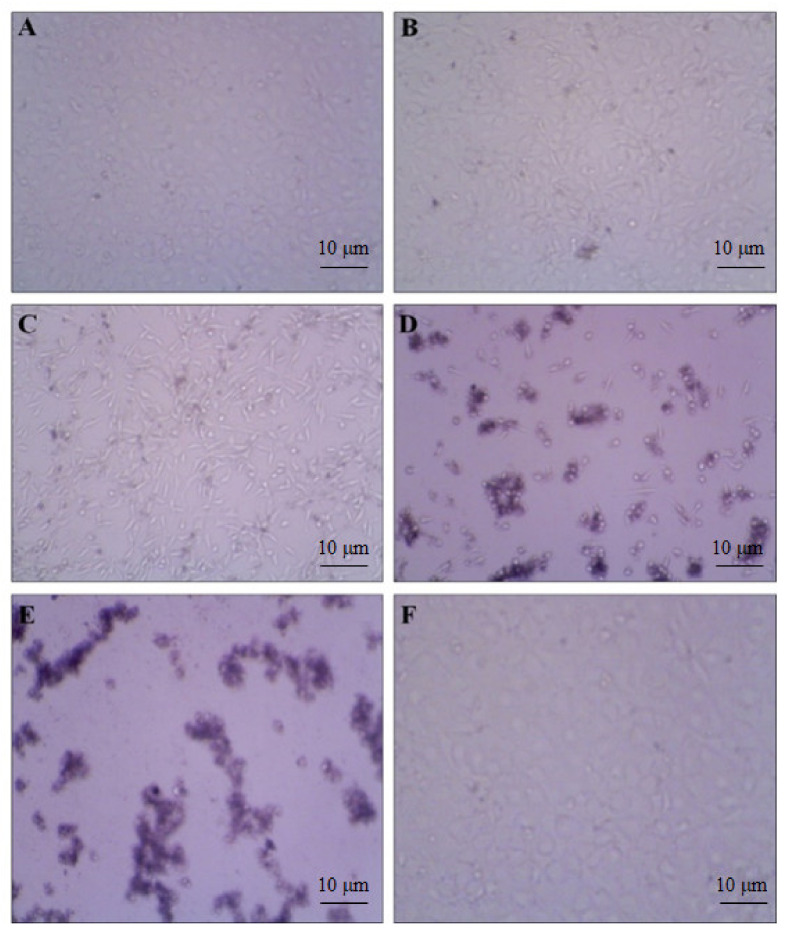
DMSO-treated control cells showed the density of the cells. (**A**) 18.75; (**B**) 37.50; (**C**) 75; (**D**) 150; and (**E**) 300 µg/mL treatment showed gradual cell toxicity compared to control. (**F**) Methanol low dose treatment. Scale bars.10µm.

To evaluate the anti-proliferative effect of our compound, we investigated the cell viability by using various concentrations (18.75, 37.5, 75, 150, and 300 µg/mL) in the HeLa cell line. We observed the increased rate of cell inhibition with increasing concentrations. Approximately 90% and 72% of cell inhibition were observed with the concentration of 300 and 150 µg/mL, respectively. The maximum inhibitory concentration (IC50) of these compounds against cervical cancer cell lines was 96 µg/mL. The cytotoxicity tests of leaf removal accomplished over half of the cell passing in a convergence of 37.5 µg/mL (Figure 7). We also observed the morphological transformation in cancer cell lines after using the various concentrations of our compound. We observed the decreased cytoplasmic ratio, which results in oval-shaped morphology in HeLa cells (Figure 8).

## 4. Discussion

Over the last decades, herbal medicinal floras for cancer therapy have gained attention due to the metabolic quality of certain organic practices in various plant compounds. Aromatic medicinal plants are a rich source of bioactive compounds with anticancer action that cause less damage in healthy cells than conventional medicine. The key goals of studying crude plant extracts were to either isolate bioactive agents for further use or to find bioactive compounds that could be used as lead compounds to study semi-artificial drugs. In our study, we have used only MeOH as a solvent because it is more used than ethanol (EtOH), the boiling point of EtOH is 78.4 °C while the boiling point of MeOH is 64.7 °C, so when you have a methanol extract, you need a lower temperature to evaporate the solvent in the rotary vapor. By this, the extract is less damaged than the EtOH one. Likewise, MeOH can extract both polar and non-polar compounds [29].

Anti-tumor effects of herbal extracts are essential for behavioral inhibition [30]. ROS have a direct relationship with the tumor since the former is elevated in the tumor. Elevated ROS are quenched by increased antioxidant enzymatically in the same tumor cells. DPPH has a hydrogen donating ability in radical-scavenging activities that are very important to prevent the deleterious role of free radicals in different diseases, including cancer. DPPH-free radical-scavenging is an accepted mechanism for screening the antioxidant activity of plant extracts [31]. Therefore, the extracts that have strong radical scavengers are naturally good antioxidants, which is consistent with the previous reports [32]. In our study, we found that *AT* has good antioxidant properties, which is consistent with the previous reports [20].

We also found out that the MeOH extract of *AT* reduces the cell growth in HeLa cells dose-dependently. It was on similar lines where MeOH extracts of *Jatropha curcus* and *J. gossypifolia* inhibited cell growth in HeLa cells at low doses. Both plant extracts had IC50s of 98.18 and 110.6 g/mL for substantial suppression of HeLa cell proliferation in vitro. These tests were performed on both HeLa and HPL cells using MTT and LDH leaks. An inhibitory effect of MeOH extract on HeLa cell growth (IC50 = 100g/mL) was found at low concentrations. To normal cells, the extract was less harmful. Because of its chemoprotective properties, the current research suggests that it may be utilized to treat cancer.

It is a natural property of medicinal plants and herbs to exhibit far stronger antioxidant activity compared to non-medicinal plants. This solid antioxidant activity is driven by significantly higher levels of phenolic compounds [33,34]. The application of an appropriate extraction method in medicinal plants, including a selection of the appropriate eluent to use these extracts in food production, is of great importance in terms of obtaining valuable compounds (including phenolic acids). This will increase the antioxidant activity of these compounds and ultimately will be beneficial for the producers and consumers.

Apart from antioxidant properties, the antiseptic effects of these compounds are also important. These effects may be attributed to the bioactive constituents’ varying chemical compositions and modes of action [35]. The distinction between Gram −Ve and Gram +Ve bacterial strains may be attributed to morphological variations between these microorganisms. G-Ve bacteria possess an outer phospholipidic membrane containing structural components of lipopolysaccharide. This makes the cell wall impermeable to lipophilic solutes, while porins provide a selective barrier of approximately 600 Da (Dalton) for hydrophilic solutes with an exclusion limit. Positive bacteria can be more vulnerable since they only have an exterior peptidoglycan coating, which is ineffective as a permeability buffer. The cell’s reaction to inflammation will result in pathological symptoms, such as compromised physiological functions [36]. The inflammatory response has been connected to the concept of various illnesses, including arthritis, stroke, and cancer. Our study finds out that the capacity of MeOH extract of *AT* to inhibit protein denaturation of albumin ranges from 16.75 ± 0.65 to 62.35 ± 0.20. It has shown promising evidence for its anti-inflammatory properties. This was only the preliminary testing where we have analyzed concentration-dependent percentage in membrane protection. The research also offers solid support that *AT* leaves can act as a potential anti-inflammatory agent in folkloric and tribal medicine. As a result, the plant may be considered a renewable source of membrane stabilizers, which can be an alternative treatment for inflammatory disorders and diseases.

To detect the target protein of small molecules, it is important to use the in silico molecular docking method. We used a small protein molecule docking web service, SwissDock, to detect the ligand-protein binding. It was found to be of great value for confirming prior fast dockings in a two-step structure-based virtual screening (SBVS) process [37]. The mobility of a drug from the administration site into the target site attributes to its physicochemical properties. Solubility is a valuable parameter in rational drug design and is expressed in MLog_P_, where P is the partition coefficient in octanol-water, which has implications on hydrophobic effect, bioavailability, and absorption, interactions with receptors, metabolism, and toxicity of the lead compounds. Similarly, the total polar surface area predicts the transport capability of the leads and shows a correlation with the human intestinal absorption, blood-brain barrier penetration, and Caco-2 monolayers permeability [38]. Furthermore, all of them pass the important ADME/T properties concerned with the blood-brain barrier, human oral bioavailability, human intestinal absorption, carcinogenicity, etc. [39]. Molecular docking and dynamics are the core part of designing and screening new bioactive molecules [40,41]. Molecular docking indicated that one of our compounds (compound 4, 7-acetyl-3a^1^-methyl- 4,14-dioxo-1,2,3a,3a^1^,4,5,5a,6,8a,9b,10,11,11a-tetradecahydro-2,5a epoxy5,6a (methanooxymethano)phenaleno[1′,9′:5,6,7]indeno[1,7a-*b*]oxiren-2-yl acetate) target EREG and is involved in cervical cancer. EREG is down-regulated in cervical cancer; because of its critical function, EREG is a good candidate for anticancer therapy. The protein–protein interaction study indicates the interacting partners of EREG, and they were EGF, TGFA, GRB2, and HRAS, and most of them regulate cervical cancer [42,43,44,45]. Since our target proteins were related to cervical cancer, we decided to use the most commonly used human cervical cancer cells (HeLa cells) lines (the first human cell line).

According to previous reports, HeLa cells that undergo shrinkage and lose interaction with neighboring cells are distinguished by changes in their morphology [46,47,48]. Previous reports stated that similar to *AT*, Jarong leaves also have cytotoxic properties, and they are due to chemical compounds, such as alkaloids, flavonoids, and terpenoids, which are shown to have anticancer effects. Flavonoids play an essential function in cancer chemoprevention and chemotherapy since it interacts with several various forms of genes and enzymes [49,50]. Our results indicated that the MeOH extract of *AT* reduced the cell growth in the HeLa cells line, and the reduction was dose-dependent. It was similar to the previously reported studies, where MeOH extracts of different medicinal plants *Jatropha curcus* and *J. gossypifolia* also inhibited cell growth in HeLa cells in similar manners [51]. The results in our study indicate that the decreased free radicals and oxidative stress by increasing antioxidants might play a role in ameliorating the DNA damage by reducing the rate of abnormal cell division and decreasing mutagenesis. It is the main reason for many antioxidant-rich plants to possess antibacterial and anticancer activity, such as *AT* [52,53]. 

## 5. Conclusions

The discovery of the well-known antioxidants in plants that might have bioactive compounds has raised the hopes for their use to prevent and treat the diseases mentioned above. The results in our study indicate that the active compound 4 (7-acetyl-3a^1^-methyl- 4,14-dioxo-1,2,3a,3a^1^,4,5,5a,6,8a,9b,10,11,11a-tetradecahydro-2,5a epoxy5,6a (methanooxymethano)phenaleno[1′,9′:5,6,7]indeno[1,7a-*b*]oxiren-2-yl acetate) of *AT* can be used as a novel bioactive natural product that may serve as a leading molecule in the pharmaceuticals for its anticancerous and antibacterial properties. However, a thorough in vivo and pharmacological analysis will shed more light on its working mechanism. Our finding paves the way for the future identification of possible therapeutic compounds in *AT* that can be developed into a novel modern drug.

## Figures and Tables

**Table 1:** Antibacterial activities of MeOH leaf extract from the *AT* (500 µg/disc).

Mean Zone of Inhibition ^a^ (mm) ^b^		
S. No	Microorganism	Methanol Extract (MeOH)
		500 µg/Disc
1	*Staphylococcus aureus*	15.3 ± 0.15
2	*Bacillus cereus*	11.4 ± 0.20
3	*Bacillus subtilis*	11.1 ± 0.10
4	*Escherichiacoli*	8.2 ± 0.05
5	*Klebsiellapneumonia*	10.0 ± 0.00
6	*Salmonella typhi*	10.0 ± 0.11

^a^—diameter of zone of inhibition (mm) including disc diameter of 6 mm, ^b^—mean of three assays; ± standard deviation; Ciprofloxacin (5 µg/disc)—antibacterial drug between 19 and 23 mm; Ketoconazole (10 µg/disc)—antifungal drug between 13 and 14 mm.

**Table 2:** ADMET and physicochemical properties of the title compounds.

S. No	Formula	MW	Aromatic Heavy Atoms	Rotatable Bonds	H-Bond Acceptors	H-Bond Donors	TPSA	XLOGP 3	Ali Log S	Ali Class	BBB	CYP1A2 Inhibitor	CYP2C19 Inhibitor	CYP2C9 Inhibitor	CYP2D6 Inhibitor	CYP3A4 Inhibitor	Bioavailability	SA
1	C27H41N3O2S2	503.76	6	12	3	2	112.04	5.52	−7.63	Soluble	No	No	Yes	No	Yes	Yes	0.55	5.07
2	C16H15N3S	281.38	15	2	2	0	57.89	4	−4.92	M. soluble	Yes	Yes	Yes	Yes	No	No	0.55	3.11
3	C27H42O4	430.62	0	0	4	1	55.76	4.83	−5.73	M. soluble	Yes	No	No	No	No	No	0.55	6.7
4	C12H14O3	206.24	6	2	3	0	35.53	1.96	−2.33	Soluble	Yes	Yes	No	No	No	No	0.55	1.99
5	C26H28O8	468.5	5	1	8	2	123.27	0.67	−2.84	Soluble	No	No	No	No	No	No	0.55	5.92

MV= 150–500 g m/mol, TPSA (total polar surface area) = 20 A^2^–130 A^2^, H-A = no. Of H-bond acceptors ≤ 10, H-D = no. of H- bond donor ≤ 5, Rotatable bonds = 0–9, Log S = 0–6, In saturation +0.25–1, no. atoms (number of atoms) = 20–70.

**Table 3:** Shortlisted compounds with their drug parameters and toxicity report.

S. No.	Formula	MW	Avian Toxicity	Biodegradation	Crustacea Aquatic Toxicity	Fish Aquatic Toxicity	Honey Bee Toxicity	Tetrahymena Pyriformis	Human Intestinal Absorption
**1**	C27H41N3O2S2	503.76	−	+	iii	−	−	1.18606	+
**2**	C16H15N3S	281.38	−	+	iii	+	−	1.0123	+
**3**	C27H42O4	430.62	−	−	iii	−	−	0.50823	+
**4**	C12H14O3	206.24	−	−	iii	+	−	0.85124	+
**5**	C26H28O8	468.5	−	+	i	+	−	1.7517	+

The remaining parameters of Lipinski’s rule, namely the “number of hydrogen bond acceptors” (NHA), number of hydrogen bond donors, and “number of rotatable bonds” (nRotB), are also crucial to evaluate drug likeliness.

**Table 4:** Drug likeness (indicated by Lipinski properties) of the selected bioactive compound of *Azima tetracantha* analyzed with Swiss ADME.

Bioactive Compounds	Lipinski’s Parameters						Mv	nRotB
	Molecular Weight	Log P	nHBA	nHBD	TPSA (A^2^)	N Violations		
(*E*)-*S-tert*-butyl 2-((*E*)-4,4-dimethyl-5-((2,3,3-trimethyl-5-(methylthio)-3,4-dihydro-2*H*-pyrrol-2-yl)methylene)pyrrolidin-2-ylidene)-2-(3,4-dimethyl-5-oxo-2,5-dihydro-1*H*-pyrrol-2-yl)ethanethioate	503.76	4.99	3	1	61.43	1	482.64	10
5,6-dimethoxy-3-methyl-2,3-dihydro-1*H*-indene-1-one	281.38	5.26	3	0	29.66	1	255.16	2
5-(4-aminophenyl)-4-(*o*-tolyl) thiazol-2-amine	430.62	5.01	4	1	57.51	1	427.78	3
2-(3-acetoxy-4,4,10,13,14 pentamethyl2,3,4,5,6,7,10,11,12,13,14,15,16,17- tetradecahydro-1*H*-cyclopenta[*a*]phenanthren-17-yl) propanoic acid	206.24	1.86	2	0	35.41	0	193.6	2
7-acetyl-3a^1^-methyl-4,14-dioxo-1,2,3a,3a^1^,4,5,5a,6,8a,9b,10,11,11a-tetradecahydro-2,5a^1^-epoxy-5,6a(methanooxymethano)phenaleno[1′,9′:5,6,7]indeno[1,7a-*b*]oxiren-2-yl acetate	468.5	0.91	8	2	123.28	0	406.2	2

All compounds passed the pre-set criteria: MLogP ≤ 5, nON ≤ 10, and nOHNH ≤ 5 with zero violation (i.e., N Violations ≤ 1) (Table 4), indicating that they may be good candidates for drug design.

**Table 5:** Docking analysis of predicted compounds from the methanol extract of *Azima tetracantha* leaf with 4AGD/2ITY Protein.

S. No.	Compound Name	4AGD		2ITY	
		Docking Score (kcal/mol)	Glide Energy (kcal/mol)	Docking Score (kcal/mol)	Glide Energy (kcal/mol)
**1**	5,6-dimethoxy-3-methyl-2,3-dihydro-1H-indene-1-one	−4.91	−26.65	−5.58	−34.33
**2**	5-(4-aminophenyl)-4-(o-tolyl)thiazol-2-amine	−4.48	−26.59	−6.80	−36.51
**3**	2-(3-acetoxy−4,4,10,13,14-pentamethyl-2,3,4,5,6,7,10,11,12,13,14,15,16,17-tetradecahydro-1H- cyclopenta[a]phenanthren-17-yl) propanoic acid	−4.47	−39.05	−6.81	−43.53
**4**	7-acetyl-3a^1^-methyl-4,14-dioxo-1,2,3a,3a^1^,4,5,5a,6,8a,9b,10,11,11a-tetradecahydro-2,5a^1^-epoxy5,6a(methanooxymethano)phenaleno[1′,9′:5,6,7]indeno[1,7a-b]oxiren-2-yl acetate	−6.47	−52.25	−5.79	−44.60

## Data Availability

Data is contained within the article and Supplementary Materials.

## Supplementary Materials

The following are available online at https://www.mdpi.com/article/10.3390/antiox10081307/s1, Figure S1: Phytocompounds identified from methanol extract of *Azima tetracantha*. Figure S2: HPLC analysis of *Azima tetracntha.*

## Abbreviation

MeOH	Methanol
*Azima tetracanta*	*AT*
ADME/T	(Absorption, Distribution, Metabolism, Excretion) and Toxicity
STRING	Search Tool for the Retrieval of Interacting Genes/Proteins
HPLC	High-Performance Liquid Chromatography analysis
MW	Molecular weight
GC/MS	Gas chromatography mass spectroscopy
KEGG	Kyoto Encyclopedia of Genes and Genomes
REATOME	Reactome pathway database
LogP	Log of octanol/water partition coefficient
nHBA	No. of hydrogen bond acceptor(s)
nHBD	No. of hydrogen bond donor(s)
TPSA	Total polar surface area
N Violations	No. of rule of five violations
MS	Molar aqueous solubility
MV	Molar volume
nRotB	No. of rotable bonds
MTT	3-(4,5-dimethylthiazol-2-yl)-2,5-diphenyl tetrazolium bromide
