# Supplementary figures and images for: In Vitro and In Silico Toxicological Properties of Natural Antioxidant Therapeutic Agent Azima tetracantha. LAM
Source: Antioxidants (Basel). 2021 Aug 18;10(8):1307. doi: 10.3390/antiox10081307 (PMC8389312; doi:10.3390/antiox10081307)

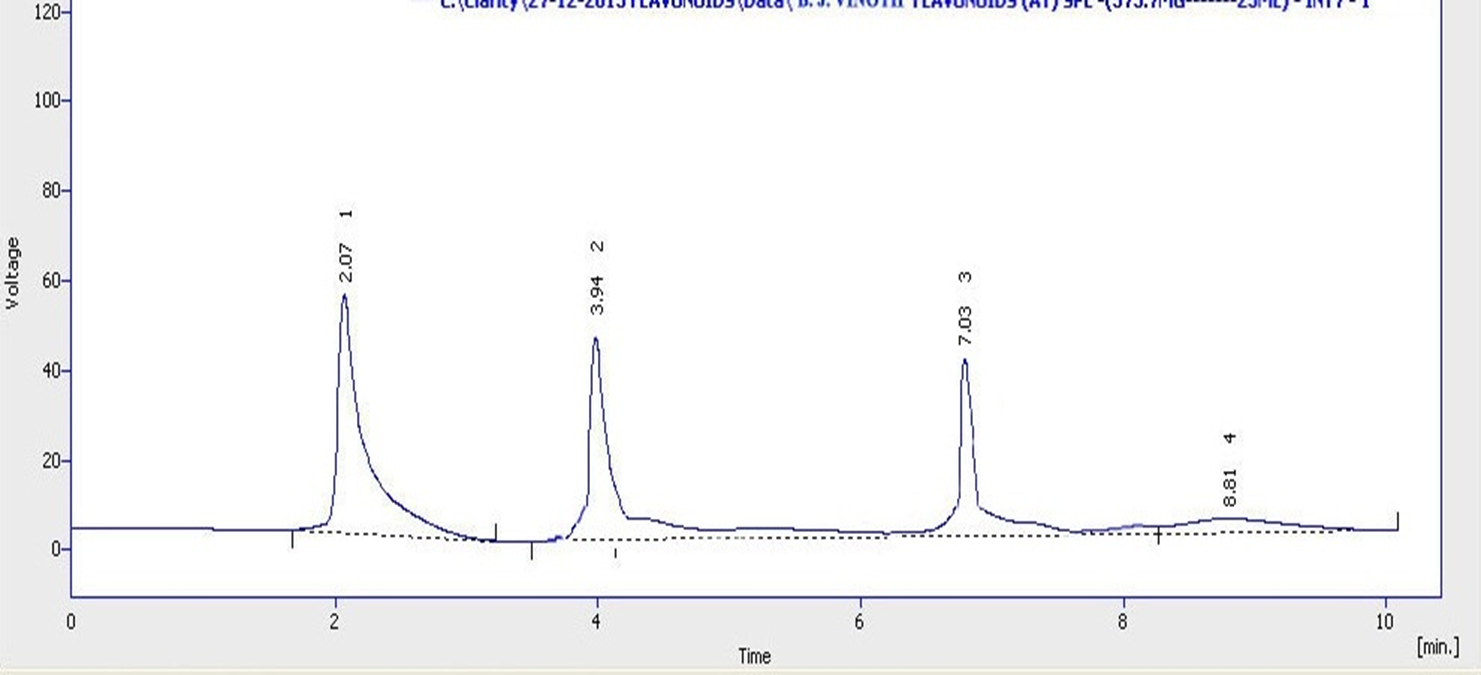

Supplement: Supplementary file 1 [file antioxidants-10-01307-s001.zip › Figure S2.png]

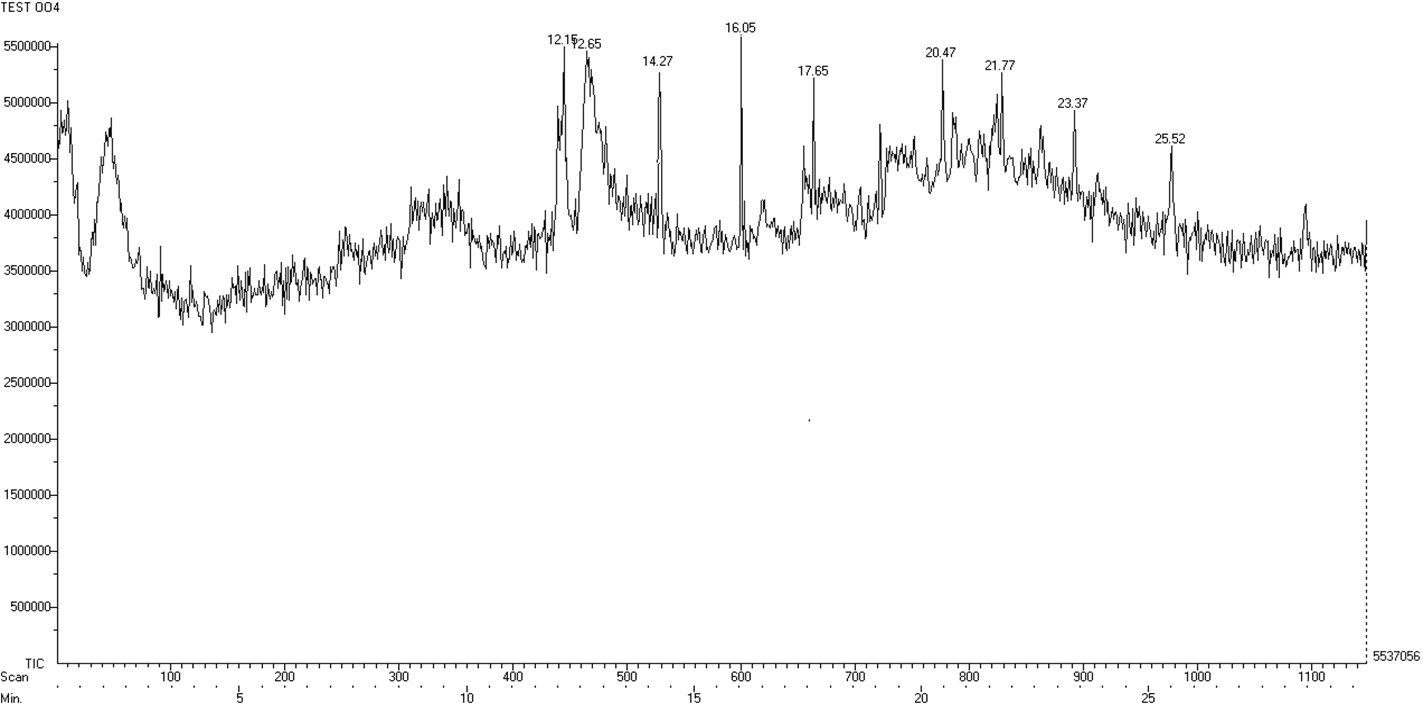

Supplement: Supplementary file 1 [file antioxidants-10-01307-s001.zip › S1.png]
